# Clinical implications of lymphadenectomy for invasive ductal carcinoma of the body or tail of the pancreas

**DOI:** 10.1002/ags3.12551

**Published:** 2022-01-18

**Authors:** Takuya Minagawa, Teiichi Sugiura, Yukiyasu Okamura, Takaaki Ito, Yusuke Yamamoto, Ryo Ashida, Katsuhisa Ohgi, Keiko Sasaki, Katsuhiko Uesaka

**Affiliations:** ^1^ 38471 Division of Hepato‐Biliary‐Pancreatic Surgery Shizuoka Cancer Center Shizuoka Japan; ^2^ 38471 Division of Pathology Shizuoka Cancer Center Shizuoka Japan

**Keywords:** dissection, lymph node station, lymphatic metastasis, pancreatic cancer, tumor location

## Abstract

**Aim:**

The appropriate extent of lymphadenectomy for pancreatic cancer of the body/tail has not been standardized worldwide. The present study evaluated the optimal extent of harvesting lymph nodes.

**Methods:**

Patients who underwent distal pancreatectomy for invasive ductal carcinoma of the pancreas between 2007 and 2018 were retrospectively reviewed. Patients were subclassified into three groups depending on the tumor location: pancreatic body (Pb), proximal pancreatic tail (Ptp), and distal pancreatic tail (Ptd). The pancreatic tail was further divided into even sections of Ptp and Ptd. Patterns of lymph node metastasis and the impact of lymph node metastasis on the prognosis were examined.

**Results:**

A total of 120 patients were evaluated. Fifty‐eight patients had a tumor in the Pb, 38 in the Ptp, and 24 in the Ptd. No patients with a Ptd tumor had metastasis beyond the peripancreatic and splenic hilar lymph nodes (LN‐PSH). All patients with metastasis to the lymph nodes along the common hepatic artery (LN‐CHA) or along the left lateral superior mesenteric artery (LN‐SMA) also had metastasis to the LN‐PSH. Recurrence after surgery occurred significantly earlier in this population. In a multivariate analysis, metastasis to the LN‐CHA or LN‐SMA (hazard ratio [HR] 3.3; *P* = .04) was an independent risk factor for overall survival. Furthermore, high levels of preoperative serum CA19‐9 (HR 10.9; *P* = .013) were a predictive factor for metastasis to the LN‐CHA or LN‐SMA.

**Conclusions:**

Metastasis to the LN‐CHA or LN‐SMA was rare but a significant prognostic factor in patients with pancreatic body/tail cancer.

## Graphical abstract

Metastasis to lymph nodes along the common hepatic artery or the superior mesenteric artery, which is a prognostic factor for patients with pancreatic body/tail cancer, was seen in no patients with tumors in the distal tail of the pancreas.

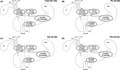

## 1 INTRODUCTION

Lymph node status is well known to be a significant prognostic marker in patients with pancreatic cancer.^1^, ^2^, ^3^, ^4^, ^5^ Pancreatectomy with lymphadenectomy has been the standard procedure for treating pancreatic cancer.^6^, ^7^ However, the optimal extent of lymphadenectomy has been controversial. Based on previous randomized controlled trials, extended lymphadenectomy with pancreatoduodenectomy has not been recommended for pancreatic head cancer.^8^, ^9^, ^10^, ^11^, ^12^, ^13^, ^14^ Particularly for patients with adenocarcinoma in the body or tail of the pancreas, few studies have focused on the influence of lymph node involvement on the prognosis.

The recommended extent of lymph node dissection during distal pancreatectomy (DP) for pancreatic cancer differs somewhat between the seventh edition of the rules of the Japan Pancreas Society (JPS)^15^ and the consensus statement by the International Study Group on Pancreatic Surgery (ISGPS).^7^ The JPS recommends harvesting lymph nodes along the common hepatic artery and the celiac axis for both pancreatic body and tail cancers. In contrast, the ISPGS recommends that the lymph nodes around the celiac axis be resected, particularly when the tumor is close to the celiac axis in the body of the pancreas, and the lymph nodes along the common hepatic artery not to be dissected for pancreatic body or tail cancers, as resection of these lymph nodes has been considered to constitute extended lymphadenectomy.^7^

Clarifying the incidence of metastasis in a specific regional lymph node station and the impact of lymph node metastasis on the prognosis has proven useful for understanding the patterns of tumor spread and examining the extent of lymph node dissection. However, to our knowledge, few studies have investigated the rates of lymph node metastasis, especially for distal pancreatic cancer.^16^, ^17^

The present study evaluated the patterns of lymph node metastasis in patients with pancreatic cancer in the body or tail and proved the validity of the current extent of lymphadenectomy during DP.

## 2 METHODS

### 2.1 Patients

From January 2007 to December 2018, 305 consecutive patients underwent DP, including 17 who underwent DP with celiac axis resection (DP‐CAR), in Shizuoka Cancer Center, Japan. Among them, 135 patients who were histologically proven to have invasive ductal carcinoma of the pancreatic body or tail were included in this study. Of these, patients who underwent R2 resection (n = 1), those who underwent DP as total remnant pancreatectomy (n = 9), and those with double cancers (n = 5) were excluded from this study. Ultimately, 120 patients were included as subjects in this study. The clinical data of these patients were obtained from a prospectively collected database.

This study was approved by the Institutional Review Board of the Shizuoka Cancer Center (approval number: J2020‐164‐2020‐1‐3).

### 2.2 Treatment strategy

Until 2012, upfront surgery was routinely performed for patients with tumors that were considered resectable. Starting in 2013, however, the surgical strategy was determined based on the resectability criteria according to the National Comprehensive Cancer Network (NCCN)^18^ and the JPS guidelines.^15^ Patients with resectable tumors received upfront surgery; those with borderline resectable (BR) tumors received neoadjuvant therapy (NAT) using chemotherapy, with or without radiotherapy (FOLFIRINOX; S‐1 + radiation; or GEM + nab‐paclitaxel [PTX]) prior to surgery; and those with locally advanced unresectable (UR‐LA) tumors received chemotherapy, with or without radiotherapy (FOLFIRINOX; GEM + radiation; or GEM + nab‐paclitaxel [PTX]). Three patients with UR‐LA underwent DP or DP‐CAR as conversion surgery. Four patients with resectable lesions received NAT followed by surgery for a clinical trial (gemcitabine [GEM] + S‐1; or S‐1 + radiation).

### 2.3 Surgical procedures

**FIGURE 1:**
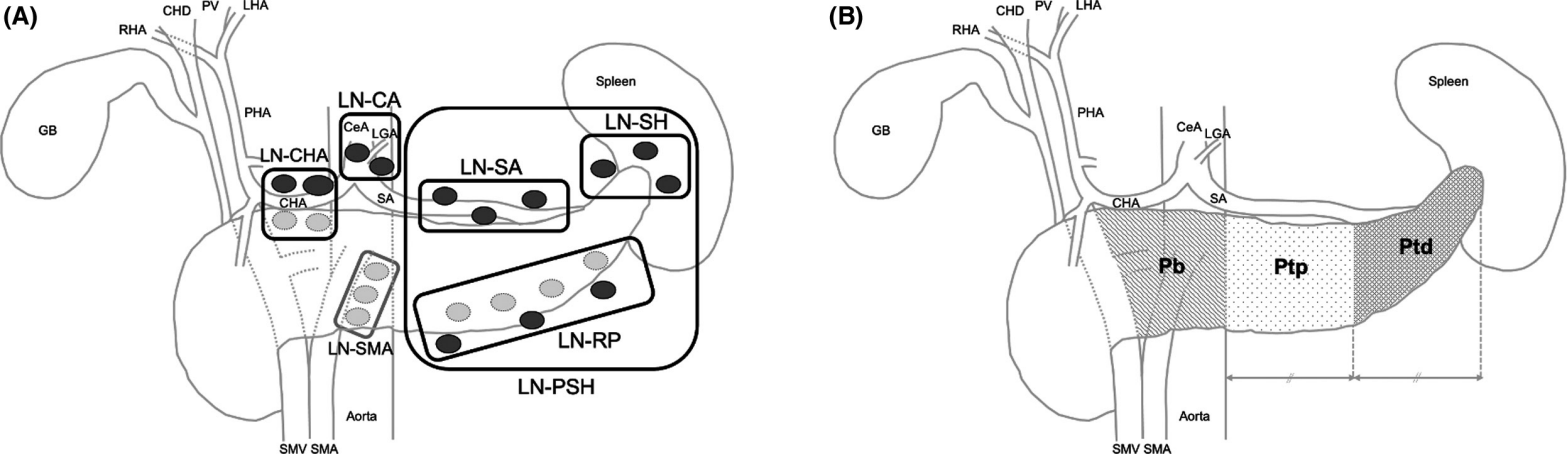
A schematic illustration of the lymph node stations dissected during distal pancreatectomy and subclassification of the tumor location. Regional lymph nodes are divided into six groups (A). Tumor location is divided into three groups (B). CeA, celiac artery; CHA, common hepatic artery; CHD, common hepatic duct; GB, gallbladder; LGA, left gastric artery; LHA, left hepatic artery; LN‐CA, lymph nodes around the celiac artery; LN‐CHA, lymph nodes along the common hepatic artery; LN‐PSH, peripancreatic and splenic hilar lymph nodes; LN‐RP, retroperitoneal lymph nodes; LN‐SA, lymph nodes along the splenic artery; LN‐SH, lymph nodes at the splenic hilum; LN‐SMA, lymph nodes along the left lateral superior mesenteric artery; Pb, pancreatic body; PHA, proper hepatic artery; Ptd, distal pancreatic tail; Ptp, proximal pancreatic tail; PV, portal vein; RHA, right hepatic artery; SA, splenic artery; SMA, superior mesenteric artery; SMV, superior mesenteric vein

All surgical procedures were performed with an open approach. No laparoscopic surgery was conducted during the study period. Peritoneal lavage cytology and sampling of the para‐aortic lymph nodes were performed after laparotomy. If unresectable factors were found, the planned procedure was abandoned. The surgical procedures performed for DP and DP‐CAR were described previously.^19^ Indications for DP‐CAR in our institution included (a) the celiac axis was involved, whereas the aorta, superior mesenteric artery, and gastroduodenal artery remained free from the tumor; or (b) preserving the splenic artery root was technically or oncologically difficult.^19^ To achieve complete lymph node dissection around the splenic artery and the splenic hilum, the spleen was routinely resected in both procedures. The extent of lymph node dissection was either equal to or greater than that recommended by the ISGPS.^7^ In detail, the lymph nodes along the common hepatic artery (LN‐CHA), around the celiac artery (LN‐CA), along the left lateral superior mesenteric artery (LN‐SMA; only in tumors in the body of the pancreas), at the splenic hilum (LN‐SH), along the splenic artery (LN‐SA), and the retroperitoneal lymph nodes (LN‐RP) were routinely dissected. A schematic image of these lymph nodes is shown in Figure 1A. The intraoperative histological evaluation of the stump of the pancreas was always performed by pathologists to ensure that the surgical margin remained negative for cancer cells.

### 2.4 Histological evaluation and numbering of lymph nodes

A histological assessment was carried out by at least two specialized pathologists. Surgeons named the lymph nodes to be dissected. Lymph nodes that had adhered to the tumor (mainly LN‐SH, LN‐SA, and LN‐RP) were not retrieved from the specimen, as the evaluation of the dissected margin would become difficult. These lymph nodes collectively included the peripancreatic and splenic hilar lymph nodes (LN‐PSH). The tumors were staged according to the eighth edition of the TNM staging manual.^20^

### 2.5 Subclassification of the tumor location

A schematic illustration of the subclassification of the tumor location is also described in Figure 1B. Tumors located at the tail of the pancreas were classified into two groups: proximal pancreatic tail (Ptp) and distal pancreatic tail (Ptd). The boundary between Ptp and Ptd was defined as the line that equally divided the left border of the abdominal aorta and the end of the pancreatic tail. If the tumor was located in more than two areas, classification was performed according to the location of the center of the tumor. Preoperative computed tomography (CT) images were used for this analysis.

### 2.6 Postoperative treatment and follow‐up

Our standard treatment for pancreatic cancer was surgery alone until 2006 and surgical resection and subsequent postoperative adjuvant chemotherapy from 2007. Adjuvant chemotherapy was conducted using gemcitabine^21^ or S‐1^22^ for 6 mo if possible. Within the first 2 y after resection, follow‐up examinations, including physical examinations, laboratory tests, assessment of tumor markers, and CT, were performed at 3‐mo intervals. If the patients had no signs of recurrence for 2 y after resection, follow‐up examinations were performed at 6‐mo intervals. The median follow‐up period of the censored patients was 22 mo.

### 2.7 Statistical analyses

Categorical variables were compared using the chi‐square test or Fisher's exact test, as appropriate. Continuous variables were compared using the Mann–Whitney *U*‐test. The survival was analyzed using Kaplan–Meier curves and the log‐rank test. The optimum cutoff values of each continuous parameter for the overall survival (OS) and predicting metastasis to the LN‐CHA or LN‐SMA were determined using the minimum *P* values calculated using the log‐rank test. Especially, as to tumor marker, cutoff values were shown to be 15.0 ng/mL for CEA (*P* = .0029) and 400 U/mL for CA19‐9 (*P* = .00047) (Figure S1A, B). Hazard ratios were estimated by univariate and multivariate survival analyses using the Cox regression model. Variables with *P* < .05 using the univariate log‐rank test were further explored in the multivariate setting. Differences were considered statistically significant at *P* < .05. All analyses were performed using the SPSS software program, v. 25.0 (IBM, Armonk, NY, USA).

## 3 RESULTS

Patients’ demographics and operative characteristics are summarized in Table 1. Fifty‐eight patients had tumors in the Pb, 38 in the Ptp, and 24 in the Ptd. Patients with tumors in the Ptd were younger than those with tumors in the Pb (*P* < .05). All patients with tumors in the Ptd had resectable lesions and underwent DP. DP‐CAR was performed in 17 patients with tumors in the Pb or Ptp. There were no other significant differences among these three groups.

**TABLE 1:** Patient characteristics stratified by tumor location

Variables	Pb	Ptp	*P* value^a^	Ptd	*P* value^b^	*P* value^c^
	(N = 58)	(N = 38)		(N = 24)		
Clinical variables						
Sex						
Male	28 (48)	23 (39)	.297	15 (63)	.332	1.000
Female	30 (52)	15 (61)		9 (37)		
Age	72 [66–77]	70 [62–77]	.198	68 [61–75]	.044*	.483
CEA (ng/mL)	2.6 [1.8–3.9]	3.0 [1.8–4.7]	.370	5.7 [2.6–17.1]	.091	.096
CA19‐9 (U/mL)	35 [10–121]	35 [11–176]	.359	77 [10–288]	.130	.915
Resectable status						
Resectable	45 (78)	35 (92)	.092	24 (100)	.008*	.277
BR/UR	13 (22)	3 (8)		0 (0)		
Neoadjuvant therapy						
No	45 (78)	34 (89)	.176	23 (96)	.056	.640
Yes	13 (22)	4 (11)		1 (4)		
Surgical variables						
Procedure						
DP	44 (76)	35 (92)	.056	24 (100)	.008*	.277
DP‐CAR	14 (24)	3 (8)		0 (0)		
Portal vein reconstruction						
No	53 (91)	36 (95)	.700	24 (100)	.315	.518
Yes	5 (9)	2 (5)		0 (0)		
Combined resected organ						
Yes	4 (7)	4 (11)	.708	6 (25)	.057	.166
Left adrenal gland	3	3		5		
Stomach	1	0		2		
Colon	0	1		3		
Others	2	0		1		
No	54 (93)	34 (90)		18 (75)		
Operation time (min)	261 [217–317]	216 [182–259]	.010*	224 [182–290]	.639	.673
Blood loss (g)	443 [264–674]	247 [145–509]	.003*	414 [241–625]	.076	.061
Pathologic variables						
Tumor size (mm)	30 [22–47]	32 [26–45]	.898	35 [25–53]	.278	.344
Tumor differentiation						
Well	22 (38)	11 (29)	.422	8 (33)	.901	.781
Mod	34 (59)	27 (71)		16 (67)		
Por	2 (3)	0 (0)		0 (0)		
pT stage (UICC 8th)						
T1	10 (17)	6 (16)	1.000	0 (0)	.107	.135
T2	29 (50)	19 (50)		15 (63)		
T3	17 (29)	12 (32)		9 (37)		
T4	2 (3)	1 (3)		0 (0)		
pN stage (UICC 8th)						
N0	27 (47)	19 (50)	.295	10 (42)	.491	.809
N1	24 (41)	18 (47)		13 (54)		
N2	7 (12)	1 (3)		1 (4)		
Number of examined LNs						
Total	16 [13–22]	18 [14–25]	.379	15 [11–18]	.076	.008*
LN‐PSH	13 [10–17]	13 [11–16]	.472	13 [10–15]	.634	.361
LN‐CA	0 [0–1]	0 [0–1]	.978	0 [0–1]	.980	1.000
LN‐CHA	2 [1–4]	2 [1–3]	.444	2 [1–5]	.637	.348
LN‐SMA	0 [0–1]	1 [0–1]	.509	0 [0–2]	.178	.448
Resection margin status						
R0	51 (88)	38 (100)	.040*	23 (96)	.426	.387
R1	7 (12)	0 (0)		1 (4)		
Postoperative variables						
AC						
No	15 (26)	7 (18)	.463	3 (13)	.247	.727
Yes	43 (74)	31 (82)		21 (87)		
AC regimen^d^						
S‐1	32 (74)	25 (81)	.586	13 (62)	.273	.196
GEM	11 (26)	6 (19)		7 (33)		
GEM + nab‐PTX	0 (0)	0 (0)		1 (5)		
First site of recurrence^e^						
Local only	7 (21)	2 (9)	.572	1 (9)	.809	1.000
Distant only	24 (73)	19 (86)		10 (91)		
Local and distant	1 (3)	1 (5)		0 (0)		
Unknown site	1 (3)	0 (0)		0 (0)		

Note

Categorical data are expressed as n (%). Continuous variables are presented as the median [interquartile range].

Abbreviations: AC, adjuvant chemotherapy; BR, borderline resectable; CA, carbohydrate antigen; CEA, carcinoembryonic antigen; DP, distal pancreatectomy; DP‐CAR, distal pancreatectomy with celiac axis resection; GEM, gemcitabine; LN, lymph node; LN‐CA, lymph nodes around the celiac artery; LN‐CHA, lymph nodes along the common hepatic artery; LN‐PSH, peripancreatic and splenic hilar lymph nodes; LN‐SMA, lymph nodes along the left lateral superior mesenteric artery; Mod, moderately; No, number; Pb, pancreatic body; Por, poorly; Ptd, pancreatic distal tail; Ptp, pancreatic proximal tail; PTX, paclitaxel; UR, unresectable.

*P < .05.

^a^ Pb vs. Ptp.

^b^ Pb vs. Ptd.

^c^ Ptp vs. Ptd.

^d^ The percentage of the total number of patients with AC is expressed in brackets.

^e^ The percentage of the total number of patients with recurrence is expressed in brackets.

No significant difference was shown in the OS and the disease‐free survival (DFS) for patients in the Pb, Ptp, and Ptd groups (Figure S2). Pathologic characteristics are also shown in Table 1. Nodal involvement was observed in 64 (53%) patients. The median number of examined regional lymph nodes was 16. R1 resection was performed in eight patients (at the pancreatic cut margin in four and at the dissected margin in four). Recurrence was observed in 66 (55%) patients during the follow‐up period. No recurrence was detected at the original area of the regional lymph nodes, although 12 patients experienced local recurrence.

### 3.1 Lymph node mapping according to the tumor location

**FIGURE 2:**
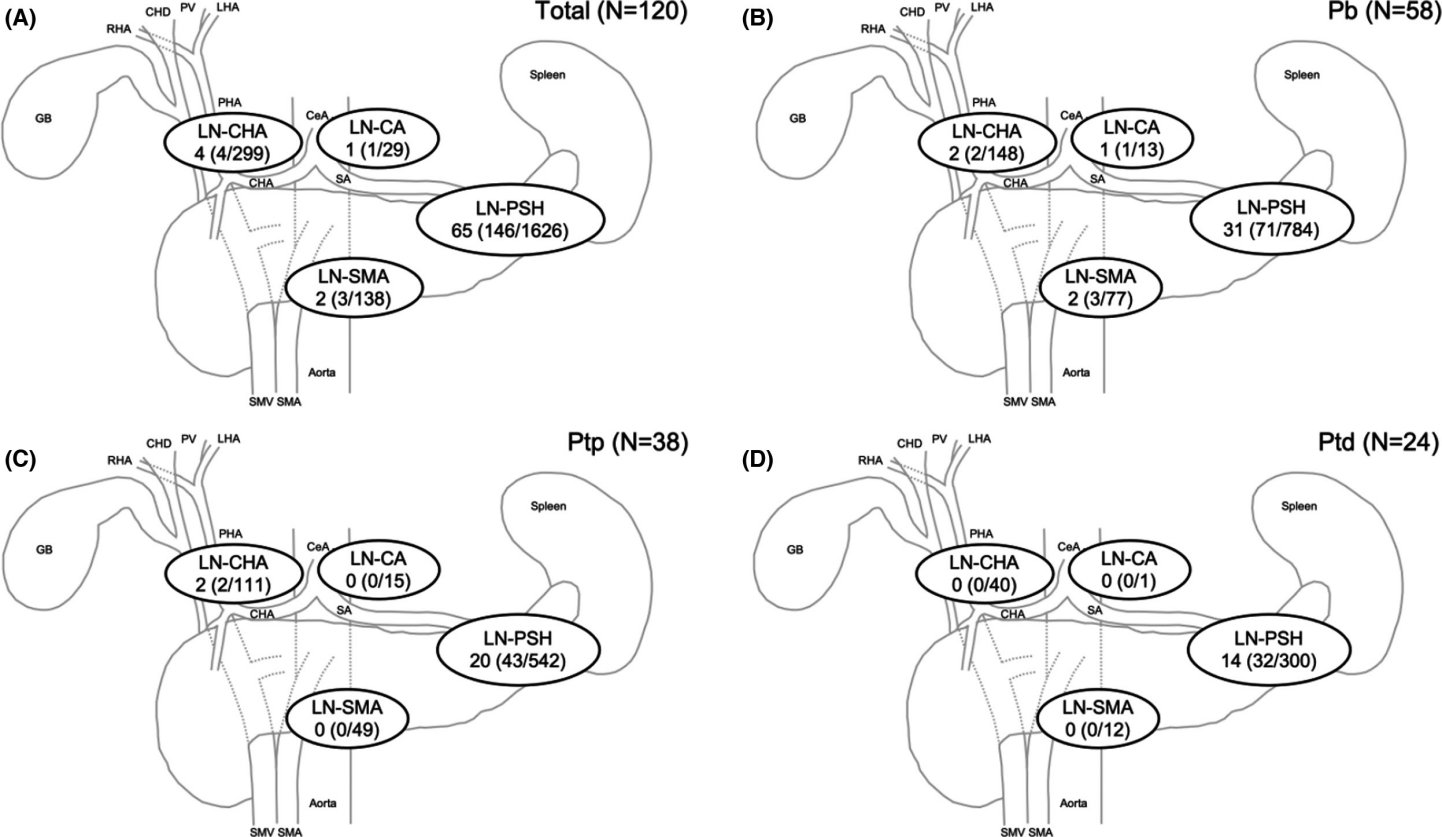
Lymph node mapping in distal pancreatectomy for pancreatic body or tail cancer. Lymph node mapping for all cases (A), pancreatic body cancer (B), pancreatic proximal tail cancer (C), and pancreatic distal tail cancer (D). Data are expressed as the number of patients with lymph node metastasis. Numbers in brackets indicate the proportion of positive lymph nodes. CeA, celiac artery; CHA, common hepatic artery; CHD, common hepatic duct; GB, gallbladder; LHA, left hepatic artery; LN‐CA, lymph nodes around the celiac artery; LN‐CHA, lymph nodes along the common hepatic artery; LN‐PSH, peripancreatic and splenic hilar lymph nodes; LN‐SMA, lymph nodes along the left lateral superior mesenteric artery; Pb, pancreatic body; PHA, proper hepatic artery; Ptd, distal pancreatic tail; Ptp, proximal pancreatic tail; PV, portal vein; RHA, right hepatic artery; SA, splenic artery; SMA, superior mesenteric artery; SMV, superior mesenteric vein

Figure 2 shows lymph node mapping in patients stratified depending on the tumor location. The most frequent metastatic lymph node was the LN‐PSH, which was attached to the pancreas. Metastasis to the LN‐PSH was observed in all patients with lymph node metastasis, regardless of tumor location. Metastasis to the LN‐CHA or LN‐SMA was observed in only four and two cases, respectively. Regarding the LN‐CA, there was only one case with a tumor in the Pb with nodal involvement. Of note, no patients with tumors in the Ptd had nodal involvement at the LN‐CHA, LN‐CA, or LN‐SMA. All patients with metastasis to the LN‐CHA or LN‐SMA had also metastasis to the LN‐PSH.

### 3.2 C**haracteristics and prognosis of patients with metastasis to the LN‐CHA or LN‐SMA**

**FIGURE 3:**
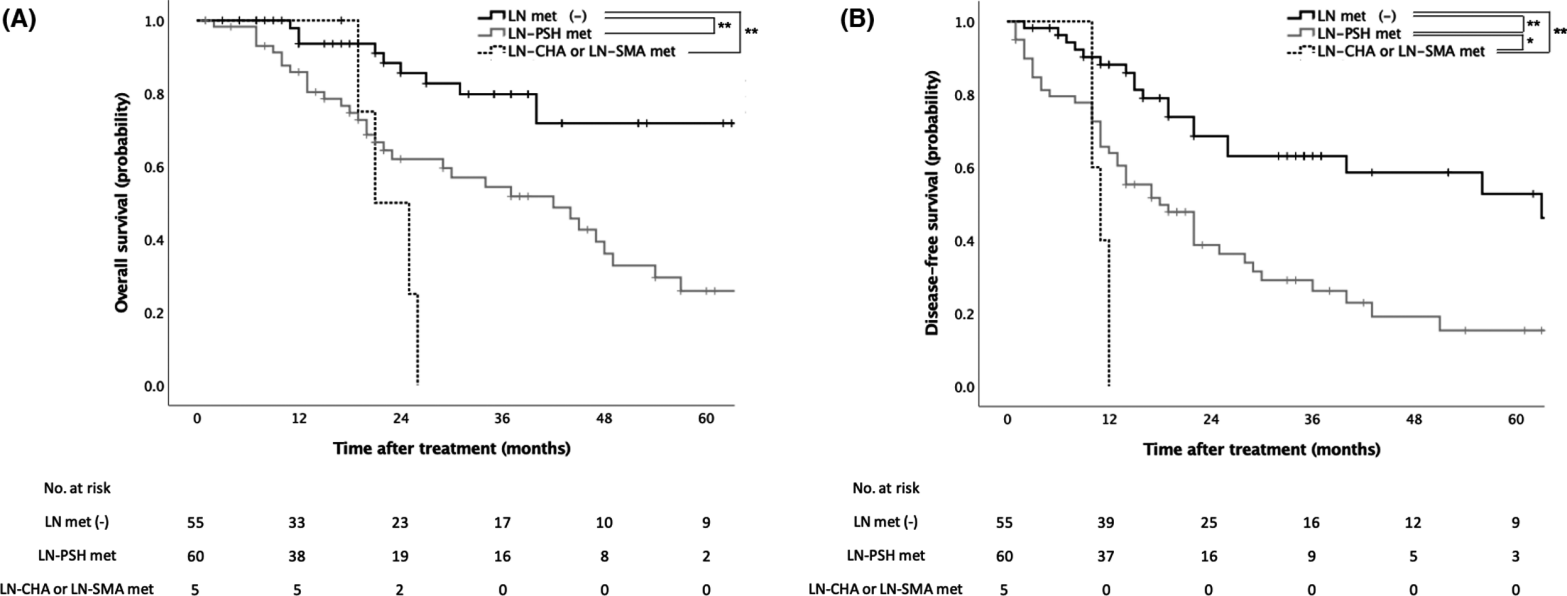
Survival analyses according to the status of lymph node metastasis. Kaplan–Meier curves for the overall survival rates (A) and disease‐free survival rates (B) of patients with no lymph node metastasis (LN met [−]), metastasis to the LN‐PSH, and metastasis to the LN‐CHA or LN‐SMA. (A) *P* < .001 (LN met [−] vs. LN‐PSH met and LN‐CHA or LN‐SMA met), *P* = .145 (LN‐PSH met vs. LN‐CHA or LN‐SMA met); (B) *P* < .001 (LN met [−] vs. LN‐PSH met and LN‐CHA or LN‐SMA met), *P* = .032 (LN‐PSH met vs. LN‐CHA or LN‐SMA met). LN, lymph node; LN‐CHA, lymph nodes along the common hepatic artery; LN‐PSH, peripancreatic and splenic hilar lymph nodes; LN‐SMA, lymph nodes along the left lateral superior mesenteric artery; met, metastasis. *P < .05, **P < .01

Early recurrence at distant organs within 1 y after surgery was observed in all cases with metastasis to the LN‐CHA or LN‐SMA (Table 2). Compared to patients with metastasis to the LN‐PSH alone, the prognosis of those with metastasis to the LN‐CHA or LN‐SMA tended to be worse (Figure 3A,B).

**TABLE 2:** Summary for patients with metastasis to LN‐CHA or LN‐SMA

Age (Y) /Sex	Tumor Location	Tumor size (mm)	CEA (mg/mL)	CA19‐9 (U/mL)	NAT	Procedure	LN‐PSH met	LN‐CHA met	LN‐SMA met	pN stage	Margin status	DFS (mo.)	OS (mo.)	Prognosis
61/M	Ptp	50	3.7	227	No	DP	Yes	Yes	No	N2	R0	12	21	Liver met, DOD
61/M	Ptp	85	3.1	1085	No	DP‐CAR	Yes	Yes	No	N1	R0	11	25	Panc/bone met, DOD
77/F	Pb	29	5.4	662	No	DP	Yes	Yes	No	N1	R0	10	19	Liver met, DOD
75/M	Pb	38	3.0	457	No	DP‐CAR	Yes	Yes	Yes	N2	R1	12	26	Pulm/Perit met, DOD
75/F	Pb	54	2.6	156	No	DP	Yes	No	Yes	N2	R0	10	17	Pulm met, AWD

Note

CEA and CA19‐9 levels were measured before surgery. pN stage was evaluated according to the 8th edition of TNM staging system.

Abbreviations: AC, adjuvant chemotherapy; AWD, alive with disease; CA, carbohydrate antigen; CEA, carcinoembryonic antigen; DFS, disease‐free survival; DOD, died of disease; DP, distal pancreatectomy; DP‐CAR, distal pancreatectomy with celiac axis resection; F, female; LN‐CHA, lymph nodes along the common hepatic artery; LN‐PSH, peripancreatic and splenic hilar lymph nodes; LN‐SMA, lymph nodes along the left lateral superior mesenteric artery; M, male; met, metastasis; mo, month; met, metastasis; NAT, neoadjuvant therapy; OS, overall survival; Panc, pancreas; Pb, pancreatic body; Perit, peritoneal; Ptp, pancreatic proximal tail; Pulm, pulmonary; Y, year.

### 3.3 Prognostic factors for OS and DFS

Multivariate analyses revealed that lymph node metastasis to the LN‐CHA or LN‐SMA, serosal invasion, portal venous system invasion, and a lack of adjuvant chemotherapy were risk factors for OS (Table 3). Similarly, a high level of serum CA19‐9, large tumor, lymph node metastasis, portal venous system invasion, and no adjuvant chemotherapy were shown to be risk factors for DFS by multivariate analyses (Table S1).

**TABLE 3:** Univariate and multivariate analyses for the overall survival

Variables	N	Median	Univariate	Multivariate	
		OS	*P* value	HR (95% CI)	*P* value
Tumor location					
Pb	58	49	.255		
Ptp	38	27			
Ptd	24	57			
Resectable status					
BR/UR	16	45	.929		
R	104	49			
CEA (ng/mL)					
≥15	8	13	.003*	1.95 (0.71–5.32)	.193
<15	112	49		1 (ref)	
CA19‐9 (U/mL)					
≥400	17	25	.001*	1.14 (0.31–4.20)	.846
<400	103	54		1 (ref)	
Neoadjuvant therapy					
No	102	47	.076		
Yes	18	NA			
Procedure					
DP‐CAR	17	26	.003*	1.35 (0.59–3.08)	.480
DP	103	NA		1 (ref)	
Portal vein reconstruction					
Yes	7	45	.693		
No	113	54			
Tumor size (mm)					
>50	26	23	.001*	1.22 (0.54–2.74)	.638
≤50	94	54		1 (ref)	
Tumor differentiation					
Mod/Por	79	47	.464		
Well	41	NA			
Lymph node metastasis					
Yes	64	34	<.001*	2.05 (0.95–4.42)	.067
No	56	NA		1 (ref)	
Metastasis to LN‐CHA or LN‐SMA					
Yes	5	21	.017*	3.30 (1.06–10.31)	.040*
No	115	49		1 (ref)	
Microscopic venous invasion					
Yes	56	42	.037*	1.17 (0.54–2.55)	.693
No	64	NA		1 (ref)	
Intrapancreatic nerve invasion					
Yes	107	47	.056		
No	13	NA			
Serosal invasion					
Yes	37	30	.005*	2.36 (1.24–4.48)	.009*
No	83	NA		1 (ref)	
Retroperitoneal invasion					
Yes	107	47	.206		
No	13	NA			
Nerve plexus invasion					
Yes	17	25	<.001*	1.33 (0.50–3.50)	.568
No	103	NA		1 (ref)	
Portal venous system invasion					
Yes	59	27	<.001*	3.05 (1.46–6.37)	.003*
No	61	NA		1 (ref)	
Arterial invasion					
Yes	33	27	.029*	1.12 (0.53–2.36)	.776
No	87	NA		1 (ref)	
Residual tumor					
Yes (R1)	8	26	.003*	1.86 (0.78–4.41)	.163
No (R0)	112	57		1 (ref)	
Adjuvant chemotherapy					
No	25	24	.001*	3.65 (1.84–7.25)	<.001*
Yes	95	57		1 (ref)	

Note

Categorical data are expressed as n (%).

Abbreviations: BR, borderline resectable; CA, carbohydrate antigen; CEA, carcinoembryonic antigen; CI, confidence interval; DP, distal pancreatectomy; DP‐CAR, distal pancreatectomy with celiac axis resection; HR, hazard ratio; LN‐CHA, lymph nodes along the common hepatic artery; LN‐SMA, lymph nodes along the left lateral superior mesenteric artery; Mod, moderately; NA, not applicable; OS, overall survival; Pb, pancreatic body; Por, poorly; Ptd, pancreatic distal tail; Ptp, pancreatic proximal tail; R, resectable; ref, reference; UR, unresectable.

*P < .05.

### 3.4 Predictive factors for metastasis to the LN‐CHA or LN‐SMA

Univariate analysis showed that high levels of preoperative serum CA19‐9 were a predictive factor for lymph node metastasis to the LN‐CHA or LN‐SMA (Table 4).

**TABLE 4:** Univariate analysis for metastasis to LN‐CHA or LN‐SMA

Variables	N	Met	Univariate	
			OR (95% CI)	*P* value
Tumor location				
Pb	58	3	1 (ref)	
Ptp	38	2	1.02 (0.16–6.40)	1.000
Ptd	24	0	0.95 (0.89–1.01)	.552
Resectable status				
BR/UR	16	0	0.95 (0.91–1.00)	1.000
R	104	5	1 (ref)	
CEA (ng/mL)				
≥15	8	0	0.96 (0.92–1.00)	1.000
<15	112	5	1 (ref)	
CA19‐9 (U/mL)				
≥400	17	3	10.82 (1.66–70.53)	.020*
<400	103	2	1 (ref)	
Neoadjuvant chemotherapy				
Yes	18	0	0.95 (0.91–1.00)	1.000
No	102	5	1 (ref)	
Procedure				
DP‐CAR	17	2	4.44 (0.69–28.83)	.146
DP	103	3	1 (ref)	
Portal vein reconstruction				
Yes	7	0	0.96 (0.92–1.00)	1.000
No	113	5	1 (ref)	
Tumor size (mm)				
>50	26	3	6.00 (0.95–38.03)	.067
≤50	94	2	1 (ref)	
Tumor differentiation				
Mod/Por	79	3	0.77 (0.12–4.80)	1.000
Well	41	2	1 (ref)	
Microscopic venous invasion				
Yes	56	1	0.27 (0.03–2.52)	.370
No	64	4	1 (ref)	
Intrapancreatic nerve invasion				
Yes	107	5	1.05 (1.00–1.09)	1.000
No	13	0	1 (ref)	
Serosal invasion				
Yes	37	0	0.94 (0.89–1.00)	.322
No	83	5	1 (ref)	
Retroperitoneal invasion				
Yes	107	5	1.05 (1.00–1.09)	1.000
No	13	0	1 (ref)	
Nerve plexus invasion				
Yes	17	1	1.55 (0.16–14.74)	.541
No	103	4	1 (ref)	
Portal vein system invasion				
Yes	59	3	1.58 (0.25–9.81)	.677
No	61	2	1 (ref)	
Arterial invasion				
Yes	33	1	0.65 (0.07–6.02)	1.000
No	87	4	1 (ref)	
Residual tumor				
Yes (R1)	8	1	3.86 (0.38–39.28)	.296
No (R0)	112	4	1 (ref)	

Note

Categorical data are expressed as n (%).

Abbreviations: CA, carbohydrate antigen; CEA, carcinoembryonic antigen; CI, confidence interval; DP, distal pancreatectomy; DP‐CAR, distal pancreatectomy with celiac axis resection; Met, metastasis; Mod, moderately; NA, not applicable; OR, odds ratio; OS, overall survival; Pb, pancreatic body; Por, poorly; Ptd, pancreatic distal tail; Ptp, pancreatic proximal tail; R, resectable; ref, reference.

*P < .05.

## 4 DISCUSSION

LN‐CHA and LN‐SMA are considered appropriate for dissection, regardless of tumor location, according to the classification of pancreatic carcinoma in Japan.^15^ However, few studies have described the metastasis rate of those stations and the effect of dissection of those lymph nodes, especially for pancreatic tail cancer.^16^, ^17^

This study describes the patterns of lymph node metastasis for patients with pancreatic body/tail cancer who underwent DP. Specifically, it revealed that LN‐CHA and LN‐SMA metastasis was rare but still a significant prognostic factor in patients with pancreatic body/tail cancer. According to the mapping of the metastatic lymph nodes, metastasis to the LN‐CHA was observed in only 3.3% of patients with tumors in the pancreatic body or proximal tail, and metastasis to the LN‐SMA was only found in 3.4% of cases in the body of the pancreas. Furthermore, no patients with tumors in the distal tail of the pancreas had metastasis to the LN‐CHA or LN‐SMA. A single cancer center in Japan reported that metastasis to the LN‐CHA was observed in only two cases (4%) among 50 patients who underwent standard DP for pancreatic cancer.^17^ Similar to our study, no patients in that study with tumors located in the tail of the pancreas had metastasis to the LN‐CHA, although one had metastasis to the LN‐SMA. These results support our own findings.

With regard to the lymphatic flow and neural invasion, the lymph nodes along the common hepatic artery to the celiac axis and at the root of the superior mesenteric artery are considered important. The present study also clarified the characteristics and prognosis of patients with metastasis to the LN‐CHA or LN‐SMA. All of them developed early recurrence at distant organs within a year after surgery. Although their prognoses were poor, they were not significantly worse than those of patients with metastasis only to the LN‐PSH, probably because of the extremely small number of subjects included in our study. A multivariate analysis revealed that metastasis to the LN‐CHA or LN‐SMA was a prognostic factor for pancreatic body/tail cancer. Furthermore, a high level of preoperative serum CA19‐9 was a predictive factor for metastasis to the LN‐CHA or LN‐SMA.

Along with these findings, the extent of lymphadenectomy might be reconsidered for patients with pancreatic body/tail cancer. Lymphadenectomy of LN‐CHA and LN‐SMA should be performed, especially for tumors in the pancreatic body or the proximal tail, as such tumors have a relatively high risk of metastasis to those lymph nodes. In contrast, retrieval of those lymph node stations might be omitted for select patients with tumors in the distal tail of the pancreas, who have a lower metastasis rate of those lymph nodes. Indeed, in cases where the LN‐CHA or LN‐SMA were not fully dissected, recurrence was not detected in those remnant lymph nodes during the follow‐up period in this study.

While surgical resection of malignant tumors with lymphadenectomy has been an integral part of the treatment for various types of cancer, advances in chemotherapy and radiation therapy have changed the concept of the role of surgery. For early‐stage stomach cancer without lymph node metastasis as a preoperative diagnosis, a low extent of lymphadenectomy has been recommended.^23^ For breast cancer without clinically lymph node metastasis, as confirmed by a sentinel node biopsy, axillary lymph node dissection has been omitted.^24^ These treatments have been supported by an accurate diagnosis for tumor staging. Regarding pancreatic cancer, in general, the concept of the sentinel lymph node hypothesis has not been adopted, and a preoperative diagnosis for staging is sometimes difficult to make, compared to cases of stomach or breast cancer. Further advances in imaging studies along with the accumulation of evidence will help resolve this issue.

The pancreatic resection line during DP is determined by considering the margin from the tumor. For tumors in the Pb or Ptp, the pancreas is often resected above the portal vein. In contrast, for tumors located only in the Ptd, the pancreas resection line can be set at the left border of the SMA. These two procedures differ in their complexity, especially with laparoscopic pancreatic resection; the procedure for tumors in the Pb or Ptp is more difficult than that for tumors in the Ptd, according to the difficulty scoring system of laparoscopic DP, which is advocated by the Japanese Society of Hepato‐Biliary‐Pancreatic Surgery.^25^ The present findings suggest that if it is acceptable to omit lymphadenectomy of the LN‐CHA and LN‐SMA for tumors in the Ptd, laparoscopic DP for those tumors may be an easier and safer procedure to perform.

The present study was associated with some limitations. First, this study had a retrospective design and was performed at a single center. Furthermore, several potential biases may have influenced the results of this study. Second, the number of lymph nodes that were evaluated was relatively small. Although we try to dissect lymph nodes according to the recommendation of the ISGPS as much as possible, in some cases the regional lymph nodes were not able to be fully dissected based on tumor‐ or patient‐related factors, which might have impaired the quality of surgery and the long‐term prognosis. In particular, the number of LN‐CA dissection procedures performed was extremely small. Several factors may have contributed to this issue: the LN‐CA was not always dissected up to the “root of the celiac artery,” and since the LN‐CA is usually resected en bloc with the LN‐SA or LN‐CHA, the LN‐CA may often be mixed into the LN‐SA or LN‐CHA when separated from the resected specimen. Thus, it is possible that the lymph nodes were not included in the tissues dissected and submitted as the LN‐CA. In addition, the numbers of metastatic LN‐CHA and LN‐SMA were extremely small, possibly due to our selection of patients for surgery. This might also be associated with our institutional policy, where the LN‐SMA is usually dissected only in cases with Pb tumors. Thus, given these potential biases, we recognize that we cannot draw any absolute conclusions from these data. To confirm the current results, a further multicenter study including data from high‐volume centers should be conducted. Nevertheless, we believe that the results of the study will help refine classical procedures.

In conclusion, metastasis to the LN‐CHA or LN‐SMA was rare but still a significant prognostic factor in patients with pancreatic body/tail cancer.

## DISCLOSURES

Funding: None.

Conflict of Interest: The authors declare no conflicts of interest for this article.

Ethical Approval: This study was approved by the Institutional Review Board of the Shizuoka Cancer Center (approval number: J2020‐164‐2020‐1‐3) and it conforms to the provisions of the Declaration of Helsinki. Informed consent was substituted by the informed opt‐out procedure because of the retrospective nature of the study, and the anonymous clinical data were used for the analysis.

## Supporting information

Appendix S1
