# Supplementary material for: Clinical implications of lymphadenectomy for invasive ductal carcinoma of the body or tail of the pancreas
Source: Ann Gastroenterol Surg. 2022 Jan 18;6(4):531–42. doi: 10.1002/ags3.12551 (PMC9271019; doi:10.1002/ags3.12551)
Supplement: Supplementary file 1 — Appendix S1 [file AGS3-6-531-s001.docx]

**Figure S1. Optimal cut off value based on the prognostic differences of the patients.** Cut off values are 15.0 ng/mL for CEA (*p* = 0.0029) (a) and 400 U/mL for CA19-9 (*p* = 0.00047) (b).

**
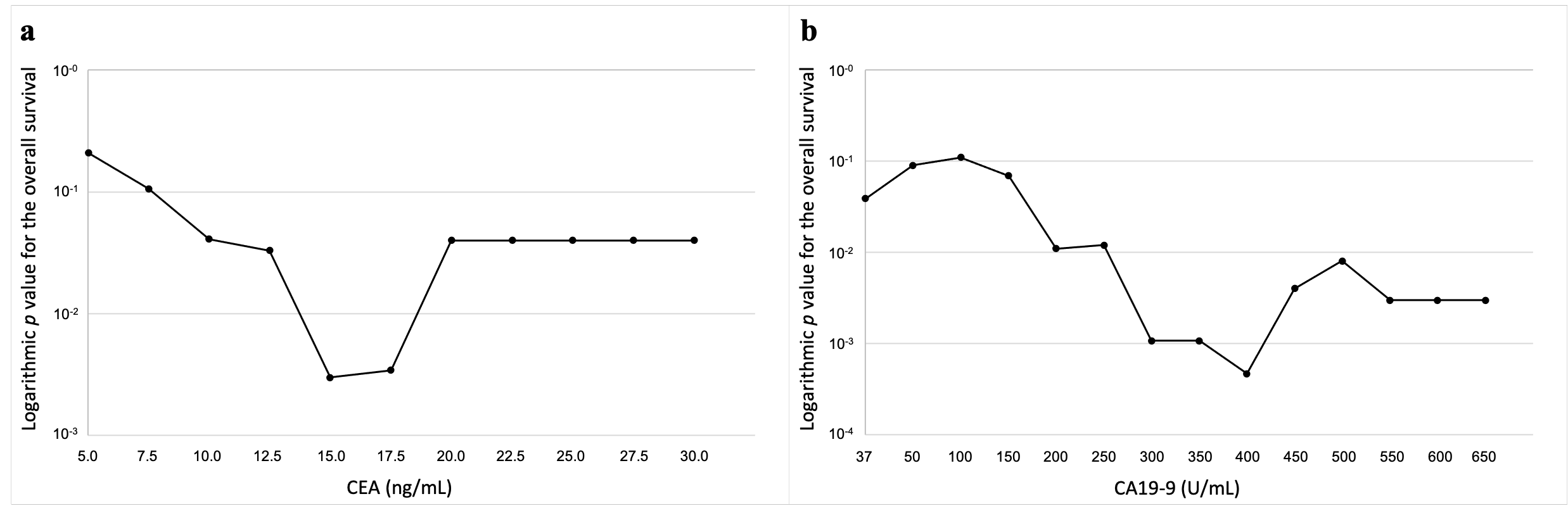
**

F**igure S2. Survival analyses according to the tumor location.** Kaplan-Meier curves for the overall survival rates (*p* = 0.255) (**a**) and disease-free survival rates (*p* = 0.472) (**b**) of patients in the Pb, Ptp and Ptd groups.

**
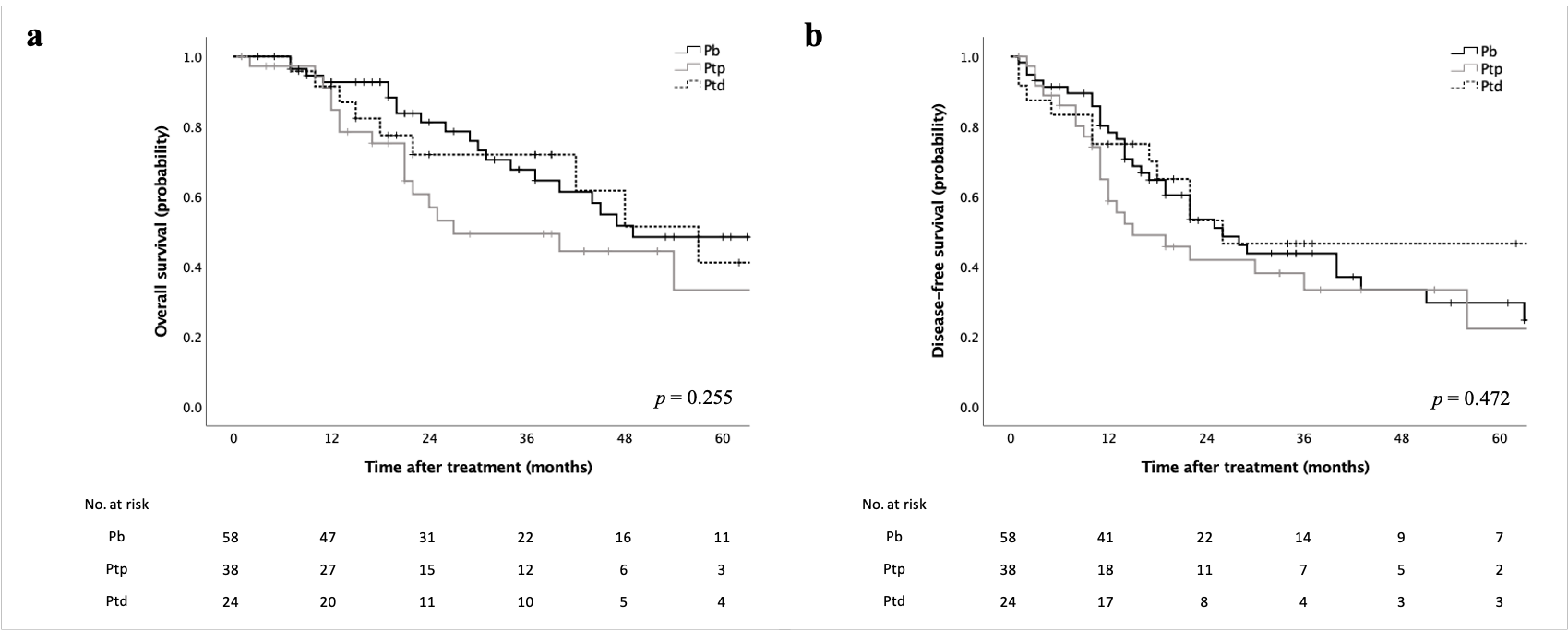
**

**Table S1. Univariate and multivariate analyses for the disease-free survival**

| Variables |  | N | Median | Univariate | Multivariate | |  |  |
| --- | --- | --- | --- | --- | --- | --- | --- | --- |
|  |  |  | DFS | *p* value | | HR (95% CI) | *p* value |  |
| Tumor location | Pb | 58 | 26 | 0.472 | |  |  |  |
|  | Ptp | 38 | 15 |  | |  |  |  |
|  | Ptd | 24 | 26 |  | |  |  |  |
| Resectable status | R | 104 | 22 | 0.726 | |  |  |  |
|  | BR/UR | 16 | 28 |  | |  |  |  |
| CEA (ng/mL) | ≥15 | 8 | 5 | <0.001* | | 1.25 (0.38-4.12) | 0.709 |  |
|  | <15 | 112 | 26 |  | | 1 (ref) |  |  |
| CA19-9 (U/mL) | ≥400 | 17 | 10 | <0.001* | | 2.49 (1.30-4.78) | 0.006* |  |
|  | <400 | 103 | 28 |  | | 1 (ref) |  |  |
| Neoadjuvant therapy | No | 102 | 22 | 0.382 |  | |  |  |
|  | Yes | 18 | 29 |  |  | |  |  |
| Procedure | DP-CAR | 17 | 14 | 0.009* | | 1.09 (0.49-2.39) | 0.837 |  |
|  | DP | 103 | 26 |  | 1 (ref) | |  |  |
| Portal vein reconstruction | Yes | 7 | 25 | 0.119 |  | |  |  |
|  | No | 113 | 22 |  |  | |  |  |
| Tumor size (mm) | >50 | 26 | 12 | <0.001* | 2.53 (1.44-4.42) | | 0.001* |  |
|  | ≤50 | 94 | 36 |  | 1 (ref) | |  |  |
| Tumor differentiation | Mod/Por | 79 | 22 | 0.124 |  | |  |  |
|  | Well | 41 | 30 |  |  | |  |  |
| Lymph node metastasis | Yes | 64 | 17 | <0.001* | 1.95 (1.09-3.48) | | 0.025* |  |
|  | No | 56 | 63 |  | 1 (ref) | |  |  |
| Metastasis to LN-CHA or LN-SMA | Yes | 5 | 11 | 0.001* | 1.86 (0.59-5.85) | | 0.292 |  |
|  | No | 115 | 26 |  | 1 (ref) | |  |  |
| Microscopic venous invasion | Yes | 56 | 17 | 0.001* | 1.43 (0.79-2.58) | | 0.241 |  |
|  | No | 64 | 56 |  | 1 (ref) | |  |  |
| Intrapancreatic nerve invasion | Yes | 107 | 22 | 0.079 |  | |  |  |
|  | No | 13 | 56 |  |  | |  |  |
| Serosal invasion | Yes | 37 | 15 | 0.024* | 1.18 (0.63-2.24) | | 0.604 |  |
|  | No | 83 | 29 |  | 1 (ref) | |  |  |
| Retroperitoneal invasion | Yes | 107 | 22 | 0.062 |  | |  |  |
|  | No | 13 | NA |  |  | |  |  |
| Nerve plexus invasion | Yes | 17 | 14 | <0.001* | 1.08 (0.53-2.22) | | 0.825 |  |
|  | No | 103 | 30 |  | 1 (ref) | |  |  |
| Portal venous system invasion | Yes | 59 | 14 | <0.001* | 2.25 (1.29-3.95) | | 0.005* |  |
|  | No | 61 | 56 |  | 1 (ref) | |  |  |
| Arterial invasion | Yes | 33 | 17 | 0.059 |  | |  |  |
|  | No | 87 | 28 |  |  | |  |  |
| Residual tumor | Yes (R1) | 8 | 10 | 0.005* | 1.83 (0.85-3.97) | | 0.125 |  |
|  | No (R0) | 112 | 26 |  | 1 (ref) | |  |  |
| Adjuvant chemotherapy | No | 25 | 10 | 0.002* | 3.16 (1.72-5.81) | | <0.001* |  |
|  | Yes | 95 | 28 |  | 1 (ref) | |  |  |

Categorical data are expressed as n (%).

* *p* < 0.05

BR, borderline resectable; CA, carbohydrate antigen; CEA, carcinoembryonic antigen; CI, confidence interval; DP, distal pancreatectomy; DP-CAR, distal pancreatectomy with celiac axis resection; HR, hazard ratio; LN-CHA, lymph nodes along the common hepatic artery; LN-SMA, lymph nodes along the left lateral superior mesenteric artery; Mod, moderately; NA, not applicable; OS, overall survival; Pb, pancreatic body; Por, poorly; Ptd, pancreatic distal tail; Ptp, pancreatic proximal tail; R, resectable; ref, reference; UR, unresectable.
